# EntropyExplorer: an R package for computing and comparing differential Shannon entropy, differential coefficient of variation and differential expression

**DOI:** 10.1186/s13104-015-1786-4

**Published:** 2015-12-30

**Authors:** Kai Wang, Charles A. Phillips, Arnold M. Saxton, Michael A. Langston

**Affiliations:** Department of Electrical Engineering and Computer Science, University of Tennessee, Knoxville, TN 37996-2250 USA; Department of Animal Science, University of Tennessee Institute of Agriculture, Knoxville, TN 37996-4574 USA

**Keywords:** Differential Shannon entropy, Differential coefficient of variation, Differential expression, Statistical tests

## Abstract

**Background:**

Differential Shannon entropy (DSE) and differential coefficient of variation (DCV) are effective metrics for the study of gene expression data. They can serve to augment differential expression (DE), and be applied in numerous settings whenever one seeks to measure differences in variability rather than mere differences in magnitude. A general purpose, easily accessible tool for DSE and DCV would help make these two metrics available to data scientists. Automated p value computations would additionally be useful, and are often easier to interpret than raw test statistic values alone.

**Results:**

*EntropyExplorer* is an R package for calculating DSE, DCV and DE. It also computes corresponding p values for each metric. All features are available through a single R function call. Based on extensive investigations in the literature, the Fligner-Killeen test was chosen to compute DCV p values. No standard method was found to be appropriate for DSE, and so permutation testing is used to calculate DSE p values.

**Conclusions:**

*EntropyExplorer* provides a convenient resource for calculating DSE, DCV, DE and associated p values. The package, along with its source code and reference manual, are freely available from the CRAN public repository at http://cran.r-project.org/web/packages/EntropyExplorer/index.html.

**Electronic supplementary material:**

The online version of this article (doi:10.1186/s13104-015-1786-4) contains supplementary material, which is available to authorized users.

## Background

Shannon entropy (SE) and coefficient of variation (CV) are used to measure the variability or dispersion of numerical data. Such variability has potential utility in numerous application domains, perhaps most notably in the analysis of high throughput biological data. Variability has been applied, for example, to study gene expression data in the context of human disease [1]. Increased entropy in particular, in both gene expression and protein interaction data, has been observed to be a characteristic of cancer [2]. Numerous other examples typify the utility of entropy [3–8] and coefficient of variation [9–12].

Shannon entropy is famously rooted in information theory [13]. To avoid confusion, we emphasize that we use the term “differential entropy” to denote a difference between two Shannon entropy values. This is distinct from information-theoretic terminology, in which “differential entropy” often means the entropy of a continuous, rather than a discrete, random variable [14].

We are particularly interested in differential analysis. In [15], we studied differential Shannon entropy (DSE) and differential coefficient of variation (DCV), and found them highly effective in identifying genes of potential interest not found by differential expression (DE) alone. DSE and DCV are applicable to other types of biological data as well, such as that produced by RNA-Seq technologies, although the usual caveats about careful interpretation apply. The usefulness of DSE and DCV is of course not limited to biological data. They may be applied to any numerical data for which normalized measures of differential variability are relevant.

### Implementation

*EntropyExplorer* is implemented in R [16]. All features are wrapped into a single function call, which takes as input up to eight arguments. Two of these arguments are numerical matrices, with identical labels for each row. The output is a matrix with two, three or five columns that contains in each row two SE, CV or mean values; a DSE, DCV or DE value; and/or two p values, one raw and one adjusted. Output rows can be sorted by value, raw p value or adjusted p value, and can be filtered to show only the top-ranked rows.

Permutation testing for DSE is accomplished with the help of the R function *sample.int*. The default number of tests to be employed is set to 1000, which the user can override. The p value for DCV is calculated by applying the Fligner-Killeen test for homogeneity of variances, implemented via the R function *fligner.test*, to the log-transform of the input data. The R function *t.test* is used to find a p value for DE. Adjusted p values are calculated using the *p.adjust* function in R, which provides false discovery rate and multiple testing corrections. A more thorough explanation of p value calculations is provided in the discussion section.

*EntropyExplorer* checks that all matrix entries are positive. This is because calculations of a DSE value/p value and a DCV p value involve taking logarithms, which are undefined on data containing zeros or negative values. Also, CV becomes less meaningful when means approach zero or are negative. Experimental data may be noisy, however, and so *EntropyExplorer* provides mechanisms to handle non-positive values. An optional two-value argument permits the user to add a positive bias to all elements of one or both matrices prior to performing any other calculations. The argument can also be set to make this adjustment automatically, based on the least non-positive value in each matrix.

### Metrics

Let $x_{1} ,x_{2} , \ldots ,x_{n}$ represent a list of *n* positive numbers, and let $x = \sum\nolimits_{i = 1}^{n} {x_{i} }$ denote their sum. The Shannon entropy of this list is

$$SE = \frac{{ - \sum\nolimits_{i = 1}^{n} {\frac{{x_{i} }}{x}\log_{2} \frac{{x_{i} }}{x}} }}{{\log_{2} n}}.$$

The coefficient of variation is

$$CV = \frac{s}{{|\bar{x}|}}$$

where $\bar{x} = x/n$ is the sample mean and $s = \sqrt {\frac{{\sum\nolimits_{i = 1}^{n} {(x_{i} - \bar{x})^{2} } }}{n - 1}}$ is the sample standard deviation. Given two such lists of positive numbers with Shannon entropies $SE_{1}$ and $SE_{2}$, coefficients of variation $CV_{1}$ and $CV_{2}$, and means $\bar{x}_{1}$ and $\bar{x}_{2}$, $DSE = |SE_{1} - SE_{2} |$, $DCV = \left| {CV_{1} - CV_{2} } \right|$, and $DE = \left| {\bar{x}_{1} - \bar{x}_{2} } \right|$.

Shannon entropy falls in the range [0, 1]; DSE therefore also falls in the range [0, 1]. Lower (higher) SE corresponds to more (less) variability. CV falls in the range [0, ∞); DCV therefore also has a range of [0, ∞).

### Application

*EntropyExplorer* is invoked as follows:

*EntropyExplorer*(*expm1, expm2, dmetric, otype, ntop, nperm*, *shift, padjustmethod*)

**Fig. 1:**
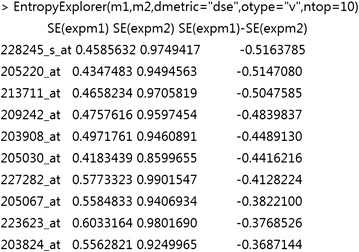
The output of *EntropyExplorer* on breast cancer data. The numerical matrices m1 and m2 have been read into R. The function call has specified “dse” for differential Shannon entropy, “v” for value, and 10 to return the top 10 values

**Fig. 2:**
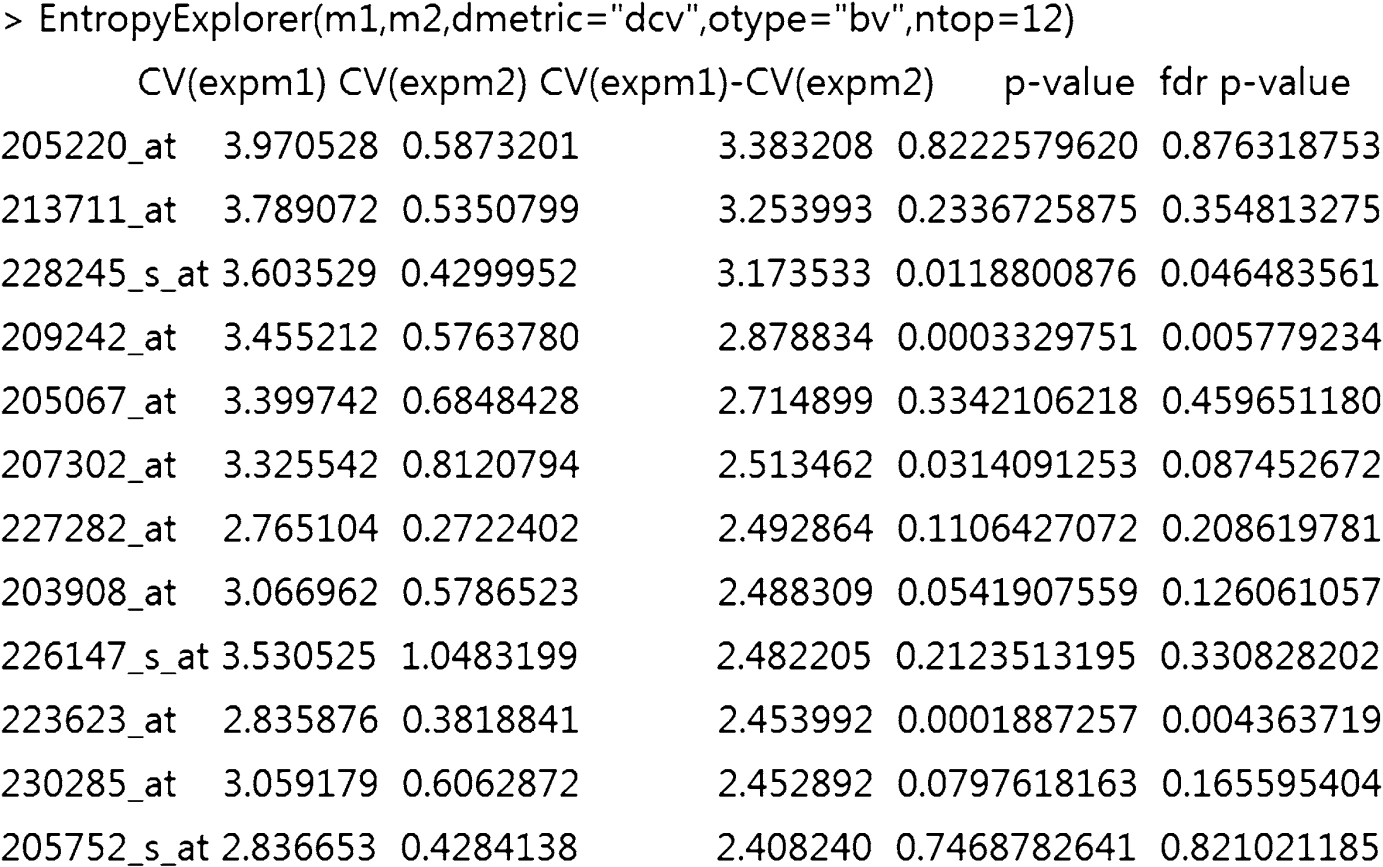
Another use of *EntropyExplorer* on breast cancer data. The function call has specified “dcv” for differential coefficient of variation, “bv” to specify both value and p value, and to sort by value, and 12 to return the top 12 rows

We refer the reader to the reference manual, included as Additional file 1 and available on the project webpage, for a detailed description of all arguments and options. Included with the package is a sample mRNA microarray dataset, consisting of a few rows from a dataset obtained from the Gene Expression Omnibus (GEO) [17]. This dataset, GSE10810, contains case/control data on breast cancer [18]. Figures 1 and 2 provide example uses of *EntropyExplorer* on the full data.

## Discussion

In addition to calculating DSE, DCV and DE, *EntropyExplorer* can calculate both raw and adjusted p values for each. ANOVA-based tests are the standard way to obtain differential expression p values. We therefore use a t-test for this purpose. Certainly more sophisticated methods exist. See, for example, [19, 20]. Thus, we emphasize that *EntropyExplorer* includes DE only as a simple, convenient and straightforward point of comparison with the other two metrics. For DCV p values, we observe that 11 tests of equal relative variation were compared in [21], with the conclusion that the Fligner-Killeen test [22] is usually the most appropriate. It strikes a balance between type I and type II errors, and is robust to non-normal distributions.

Obtaining reliable p values for DSE proved much more challenging. We found no known method in the literature specific to DSE p values. We therefore investigated the extent to which SE is correlated to variance. A high correlation would suggest that they may be proxies for each other, in which case the p value of an F-test or some derivation thereof might serve as suitable estimate of the DSE p value. Unfortunately, correlations between SE and variance, or between SE and a function of variance, were not high enough to justify using one as a surrogate for the other. Table 1 shows the correlation between SE and variance *V*, and between SE and the function $\frac{1}{2}\ln \left( {2\pi eV} \right)$ as an attempt to linearize the relationship, using the 16 datasets from [15]. The only notably high correlation is found in the obesity dataset. The obesity data, however, contains a large number of missing values, rendering the high correlation less reliable. We conclude that standard statistical tests related to variance do not appear suitable for testing DSE.

**Table 1:** Correlations between SE and variance, and between SE and $\frac{1}{2}\ln \left( {2\pi eV} \right)$, on 16 microarray gene expression datasets

Datasets	Correlation Between SE and Variance		Correlation between SE and $\frac{1}{2}\ln \left( {2\pi eV} \right)$	
	Case	Control	Case	Control
Allergic Rhinitis	−0.5515	−0.5769	−0.9703	−0.9658
Asthma_GSE4302	−0.4272	−0.4677	−0.1924	−0.2004
BreastCancer_GSE10810	−0.3942	−0.3378	−0.1810	−0.1265
CLL_GSE8835	0.2251	0.2522	−0.0806	−0.0624
ColorectalCancer_GSE9348	0.3122	0.4454	−0.0086	0.0206
CrohnsDisease_GSE6731	−0.2826	−0.2380	−0.1664	−0.4020
LungAdenocarcinoma_GSE7670	0.0725	0.3360	−0.0173	0.0105
MS_GDS3920	−0.3615	−0.3320	−0.0515	−0.0559
Obesity_GSE12050	0.9998	0.9990	0.1584	0.5420
Pancreas_GDS4102	−0.4137	−0.4455	−0.1331	−0.0890
ParkinsonsDisease_GSE20141	−0.1732	−0.2554	−0.0024	−0.0155
ProstateCancer_GSE6919_GPL8300	0.2118	0.1552	−0.0562	−0.0699
Psoriasis_GSE13355	−0.6386	−0.6554	−0.5200	−0.6779
Schizophrenia_GSE17612	0.3632	0.3910	0.0170	0.0235
T2D_GSE20966	−0.6006	−0.5550	−0.4356	−0.4663
UlcerativeColitis_GSE6731	−0.3112	−0.2555	−0.1799	−0.1451

**Fig. 3:**
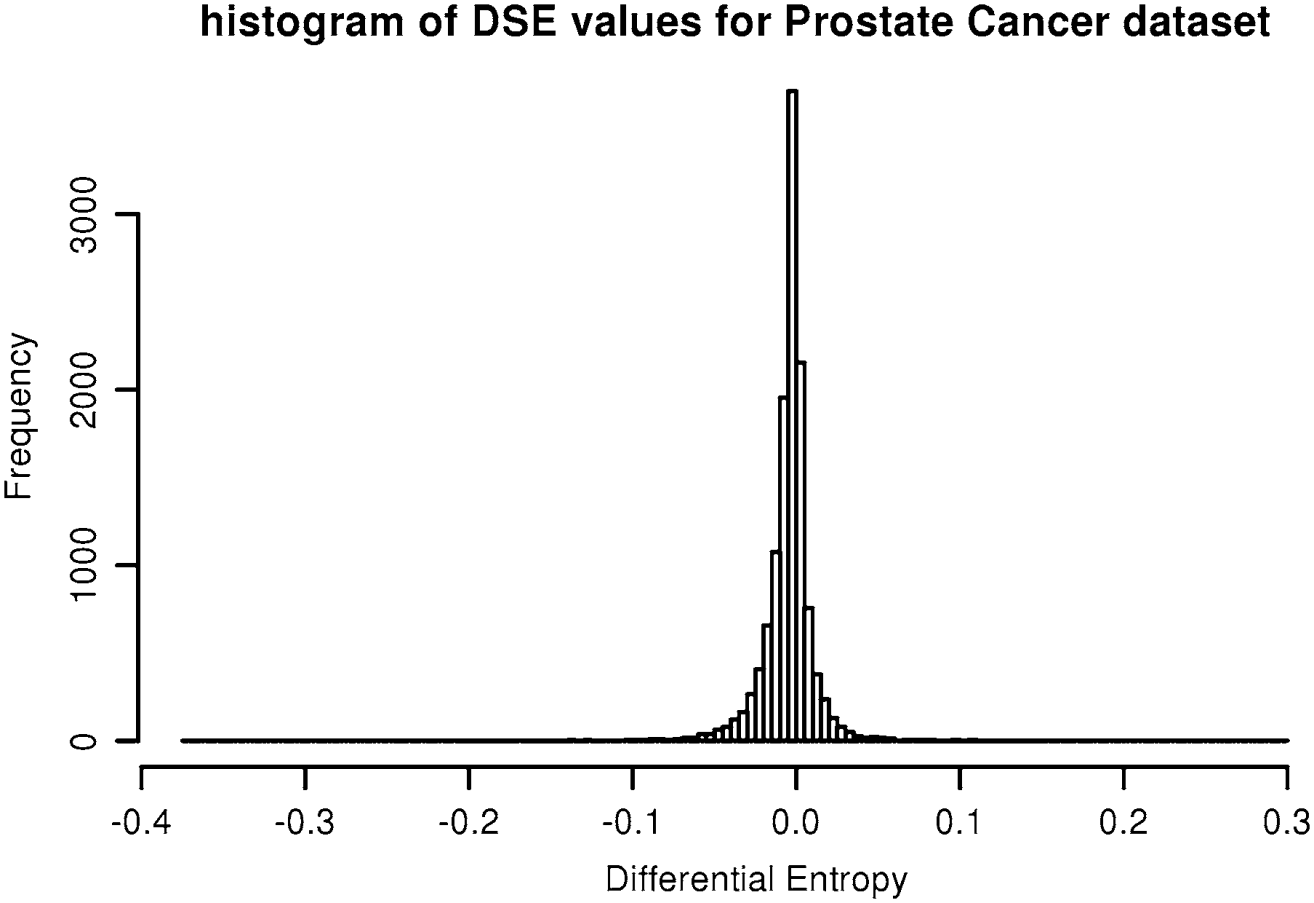
The distribution of differential Shannon entropy. The observed distribution of differential Shannon entropy in sample prostate cancer data is shown. Similar patterns were seen in all 16 data sets. None of the standard distributions tested matched the observed distributions closely enough to be considered as a reference distribution for obtaining p values

We also examined the distribution of DSE on the 16 datasets, with the goal of empirically determining a suitable reference distribution for DSE. From this, we could then estimate p values analytically. We applied the Kolmogorov–Smirnov (KS) test to compare the DSE distribution of each dataset to some of the more common reference distributions, such as normal, F, t, and Chi square. When performing a KS test, p values can be overly sensitive to deviations from the reference distribution [23], so a D-statistic value below 0.1 was used to identify matching distributions. In our experiments, only the Parkinson’s dataset produced a D-statistic below 0.1 when tested against a normal or standardized t distribution (Table 2). Figure 3 shows a sample distribution of DSE, in this case using prostate cancer data.

**Table 2:** KS test D-statistic results comparing the DSE distribution against several common distributions

Dataset	Distribution				
	Normal	Chi-square	F	t	t (standardized DSE)*
Allergic Rhinitis	0.3109	1	1	0.4991	0.3526
Asthma_GSE4302	0.2795	1	1	0.4895	0.3117
BreastCancer_GSE10810	0.2115	1	1	0.4797	0.3944
CLL_GSE8835	0.1506	1	0.9975	0.4519	0.1596
ColorectalCancer_GSE9348	0.1232	1	0.9994	0.4514	0.2142
CrohnsDisease_GSE6731	0.2131	1	0.987	0.4691	0.2392
LungAdenocarcinoma_GSE7670	0.19	1	0.9999	0.4663	0.332
MS_GDS3920	0.2703	1	0.9994	0.4813	0.3397
Obesity_GSE12050	0.2352	1	0.9991	0.484	0.287
Pancreas_GDS4102	0.2606	1	0.9937	0.4532	0.3254
ParkinsonsDisease_GSE20141	0.0628	1	0.9361	0.3816	0.0582
ProstateCancer_GSE6919_GPL8300	0.1575	1	1	0.4739	0.2522
Psoriasis_GSE13355	0.3327	1	0.9999	0.4932	0.4195
Schizophrenia_GSE17612	0.183	1	0.9998	0.4705	0.2138
T2D_GSE20966	0.3271	1	0.9999	0.4936	0.3562
UlcerativeColitis_GSE6731	0.2397	1	0.998	0.4831	0.3608

* The last column shows the results after first standardizing DSE by dividing each DSE by the standard deviation of all DSEs

We conclude from this that none of the distributions tested are close enough approximations to the observed DSE distribution to be used as a proxy for obtaining p values. Thus, without a known distribution function or suitable surrogate, we resort to resampling in order to obtain reliable DSE p values. While computationally demanding, the following permutation test makes no assumptions about the underlying distribution of the data. Given two lists of numbers, containing *n*_1_ and *n*_2_ numerical elements respectively, we first calculate their DSE and then create a new list *A* containing all $n_{1} + n_{2}$ numbers from the two lists. Next we randomly permute the elements of *A,* then recalculate DSE, treating the first $n_{1}$ elements of *A* as one list and the last $n_{2}$ elements of *A* as a second list. The resultant p value is simply the proportion of all recalculated DSEs that are at least as extreme as the original DSE.

In addition to raw p values, *EntropyExplorer* also calculates p values adjusted for multiple testing. A user can choose to adjust based on FDR, Holm or another multiple-testing adjustment.

## Conclusions

We have produced *EntropyExplorer*, an R package for calculating differential Shannon entropy, differential coefficient of variation and differential expression. This package also calculates raw and adjusted p values for each metric. These measures have been shown to complement one another [15], making this package an effective tool for users in search of more expansive suites of differential analysis methods.

## Availability and requirements

Project name: *EntropyExplorer.*

Project home page: http://cran.r-project.org/web/packages/EntropyExplorer/index.html.

Operating system(s): Platform independent.

Programming language: R.

Other requirements: R version 3.0 or later is recommended.

License: GNU General Public License version 3.0 (GPLv3).

Any restrictions to use by non-academics: None.

Additional availability: *EntropyExplorer* is integrated into the GrAPPA toolkit at http://grappa.eecs.utk.edu/.

## Additional file

10.1186/s13104-015-1786-4 EntropyExplorer R package reference manual.
