# Supplementary material for: EntropyExplorer: an R package for computing and comparing differential Shannon entropy, differential coefficient of variation and differential expression
Source: BMC Res Notes. 2015 Dec 30;8:832. doi: 10.1186/s13104-015-1786-4 (PMC4696313; doi:10.1186/s13104-015-1786-4)
Supplement: Supplementary file 1 — 10.1186/s13104-015-1786-4 EntropyExplorer R package reference manual. [file 13104_2015_1786_MOESM1_ESM.pdf]

Package ‘EntropyExplorer’

June 11, 2015

Type Package

Title Tools for Exploring Differential Shannon Entropy, Differential Coefficient of Variation and Differential Expression

Version 1.1

Date 2015-6-11

Author Kai Wang, Charles A. Phillips, Arnold M. Saxton and Michael A. Langston

Maintainer Kai Wang <kwang11@eecs.utk.edu>

Depends R (>= 3.0)

Description Rows of two matrices are compared for Shannon entropy, coefficient of variation, and expression. P-values can be requested for all metrics.

License GPL-3

NeedsCompilation no

Repository CRAN

Date/Publication 2015-06-11 21:46:55

R topics documented:

EntropyExplorer 1
GEO BreastCancer 5

Index **7**

`EntropyExplorer` *Differential Shannon Entropy, Differential Coefficient of Variation and Differential Expression*

Description

Function **EntropyExplorer** calculates differential Shannon entropy, differential coefficient of variation and differential expression, plus corresponding p-values and adjusted p-values, on two numeric matrices with identically labeled rows.

Usage

```
EntropyExplorer(expm1, expm2, dmetric, otype, ntop, nperm, shift, padjustmethod)
```

Arguments

`expm1`	A numeric matrix with labeled rows and columns.
`expm2`	A numeric matrix with labeled rows and columns. The number of rows and the row labels must exactly match those in *expm1*. The order of *expm1* and *expm2* does not matter.
`dmetric`	Specifies which differential metric to calculate. It must be either "de" for differential expression, "dse" for differential Shannon entropy or "dcv" for differential coefficient of variation. The formulas used to calculate differential Shannon entropy and differential coefficient of variation appear in [1].
`otype`	Specifies the output type, either "v" for value, "pr" for p-value with output sorted by raw p-value, "pa" for p-value with output sorted by adjusted p-value, "bv" for both value and p-value with output sorted by absolute differential value, "br" for both value and p-value with output sorted by raw p-value, "ba" for both value and p-value with output sorted by adjusted p-value, "vu" for value without sorting the output, "pu" for p-value without sorting the output, or "bu" for both value and p-value without sorting the output. The particular calculation or statistical test performed on each row of the two input matrices is based on which combination of *dmetric* and *otype* arguments is specified. The table below describes the possible combinations.

dmetric	*otype*	output
de	v	Difference between mean expression levels
de	pr, pa	Differential expression p-value obtained from a t-test and adjusted p-value, sorted by raw or adjusted p-value.
dse	v	Differential Shannon entropy
dse	pr, pa	Differential Shannon entropy p-value obtained from a permutation test and adjusted p-value, sorted by raw or adjusted p-value.
dcv	v	Differential coefficient of variation
dcv	pr, pa	Differential coefficient of variation p-value obtained from the Fligner-Killeen test, as suggested in [2] and adjusted p-value, sorted by raw or adjusted p-value.
de,dse,dcv	bv	Both values and raw and adjusted p-values, sorted by absolute value
de,dse,dcv	br, ba	Both values and raw and adjusted p-values, sorted by raw or adjusted p-value
de,dse,dcv	vu	Differential values, unsorted
de,dse,dcv	pu	Differential raw and adjusted p-values, unsorted
de,dse,dcv	bu	Both values and raw and adjusted p-values, unsorted

`ntop`	Optional argument specifying the number of top genes/probe IDs to return. If set to 10, for example, the function will return the 10 genes/probe IDs with the most significant values, or the first 10 genes/proble IDs in the input if unsorted output is requested. It must be less than or equal to the number of rows in the input data. The default is to return values for all rows in the data set.
`nperm`	Optional argument specifying the number of permutations to use to obtain a

significance p-value. It is only used when *dmetric*="dse" and *otype*="pr", "pa", "pu", "br", "ba", "bv", or "bu". The default is 1000. Higher values will yield more precise p-values, but will require longer runtimes. Values lower than 1000 will yield less precise p-values and are not recommended. On data with tens of thousands of rows, permutation testing can take up to several hours.

`shift` Optional two-value argument for use when one or both of *expm1* and *expm2* contain at least one non-positive value. The first value is added to each element of *expm1*, and the second value is added to each element of *expm2*. If the first value is "auto", and *expm1* contains non-positive values, then v_1 + .001 is added to each value in *expm1* prior to any other calculations, where v_1 is the absolute value of the smallest non-positive number in *expm1*. Likewise, if the second value is "auto", and *expm2* contains non-positive values, then v_2 + .001 is added to each value in *expm2* prior to any other calculations, where v_2 is the absolute value of the smallest non-positive number in *expm2*.

`padjustmethod` Correction method for p-values. It can be one of "holm", "hochberg", "hommel", "bonferroni", "BH", "BY", "fdr", or "none". The default is "fdr".

Details

The package is intended for use on two numeric matrices that have corresponding rows with identical labels. One example of such data is case/control microarray expression data. An example data set of this type is provided with the package. Other types of data for which the package is applicable include two-case experiments, where two groups are subjected to different stimuli, and data involving measurement at two different time points. The package consists of one function, **EntropyExplorer**. It calculates one of differential expression, differential Shannon entropy, differential coefficient of variation, or a p-value and adjusted p-value for one of the three. By default, **EntropyExplorer** returns results for every row in the data. An optional argument can be used to return only the most significant *ntop* rows or the first *ntop* rows in the input. If a row has fewer than four expression values in either matrix, then no meaningful value will be reported for that row. When both matrices have identical values on the same row and differential expression p-values are requested, then no meaningful p-values will be reported for that row. When these two situations occurs, **EntropyExplorer** will output a warning reporting the number of excluded rows.

Value

The function returns a two, three or five-column matrix, either sorted so that the most significant values are first, or unsorted so that the rows are displayed in the input order. The signs of the differential values are displayed to show direction of change between the two inputs. The matrix contains either *ntop* rows or the number of rows in the input data if *ntop* is not specified.

Note

The arguments *expm1* and *expm2* should be numeric matrices where the row IDs between the two matrices correspond. The examples below assume a simple tab-separated file format, with row and column labels included, but with no other information, such as meta-data, headers or footers. If different formatting is used, such as using spaces or commas as separators, then the commands below should be altered to reflect the particular format. File formats such as Excel spreadsheets should use the appropriate R commands to read the files into matrices.

Note that using arguments *dmetric*="dse" and *otype*="pr", "pa", "pu", "bv", "br", "ba" or "bu" will often take considerably longer than other arguments, because permutation testing can be computationally demanding. As a rough benchmark, using the default of 1000 permutations, a runtime of an hour on data with 20k rows is fairly typical.

We recommend using R version 3.0 or later with this package. Older versions may experience unanticipated problems.

Author(s)

Kai Wang, Charles A. Phillips, Arnold M. Saxton and Michael A. Langston

Maintainer: Kai Wang <kwang11@eecs.utk.edu>

References

[1] Kai Wang, Charles A. Phillips, Gary L. Rogers, Fredrik Barrenas, Mikael Benson, Michael A. Langston (2014) Differential Shannon entropy and differential coefficient of variation: alternatives and augmentations to differential expression in the search for disease-related genes. International Journal of Computational Biology and Drug Design 7(2-3):183-194

[2] Donnelly, S. M. and Kramer, A. (1999) Testing for multiple species in fossil samples: an evaluation and comparison of tests for equal relative variation. American Journal of Physical Anthropology 108(4):507-529

Examples

```
#These examples use a small subset of mRNA microarray expression data
#obtained from the Gene Expression Omnibus. The files BreastCancer_case_10.txt
#and BreastCancer_control_10.txt are in tab-separated format and
#have been stripped of all metadata, header and footer information. They contain
#the row labels (probe IDs) and column labels (samples).

#Read the two files into matrices
#When reading your own data into memory, replace casefile and controlfile
#with the complete pathname of your two data files
casefile <- system.file("extdata", "BreastCancer_case_10.txt", package = "EntropyExplorer")
controlfile <- system.file("extdata", "BreastCancer_control_10.txt", package = "EntropyExplorer")

m1<-as.matrix(read.table(file=casefile,header=TRUE, sep="\t",row.names=1))
m2<-as.matrix(read.table(file=controlfile,header=TRUE, sep="\t",row.names=1))

#calculate the difference between mean expression levels for each row
#and sort output by absolute differential values
EntropyExplorer(m1, m2, "de", "v")

#perform a t-test to calculate differential expression (adjusted) p-value
#for each row; return only the 5 most significant raw p-values
EntropyExplorer(m1, m2, "de", "pr", 5)

#calculate the value and (adjusted) p-value for differential Shannon
#entropy for each row using a permutation test with default number of
#permutations; return only the 4 largest by absolute values
```

```
EntropyExplorer(m1, m2, "dse", "bv", 4)

#calculate the value and (adjusted) p-value for differential Shannon
#entropy for the first 4 rows in the input using a permutation test
#with default number of permutations and "holm" p-value adjustment method
EntropyExplorer(m1, m2, "dse", "bu", 4, padjustmethod="holm")

#Calculate the value and (adjusted) p-value for differential Shannon
#entropy for each row using a permutation test with 1100 permutations,
#sort the output by adjusted p-value
EntropyExplorer(m1, m2, "dse", "ba", nperm=1100)

#Calculate the (adjusted) p-value for differential Shannon entropy
#for each row using a permutation test with 1050 permutations;
#return only the 3 most significant raw p-values
EntropyExplorer(m1, m2, "dse", "pr", 3, 1050)

#Calculate the differential coefficient of variation (adjusted) p-value for
#each row using the Fligner-Killeen test
EntropyExplorer(m1, m2, "dcv", "pr")

#Calculate the differential coefficient of variation for each
#row; return the 8 most significant by absolute values
EntropyExplorer(m1, m2, "dcv", "v", 8)

#Add 2.3 to every value in m1 and 2.8 to every value in m2, then
#calculate the differential Shannon entropy for each row;
#return the 6 most significant by absolute values
EntropyExplorer(m1, m2, "dse", "v", 6, shift=c(2.3, 2.8))

#Add an amount to each value in  m1 equal to the absolute
#value of the smallest non-positive number in m1 plus .001.
#Add an amount to each value in m2 equal to the absolute
#value of the smallest non-positive number in m2 plus .001.
#Calculate the differential coefficient of variation and DCV (adjusted)
#p-values for each row, sort output by absolute value, return only
#the 7 most significant by absolute values
EntropyExplorer(m1, m2, "dcv", "bv", 7, shift=c("auto","auto"))
```

GEO BreastCancer *Subset of GEO GSE10810 Breast Cancer Dataset*

Description

This dataset consists of the first 10 rows of a case/control mRNA microarray dataset on human breast cancer. The intent is to provide a small dataset that helps a user quickly become familiar with the capabilities of EntropyExplorer, while at the same time using actual experimental data in a standard format. The dataset is divided into files "BreastCancer_case_10.txt" and "BreastCancer_control_10.txt" that contain 31 tumor and 27 control samples, respectively. Some of the samples are paired. The full dataset and its description are available from the Gene Expression

Omnibus at http://www.ncbi.nlm.nih.gov/geo/ [1], accession number GSE10810 [2]. The data were obtained using the Affymetrix Human Genome U133 Plus 2.0 Array, and contain mRNA expression values for more than 18k probes.

Source

Gene Expression Omnibus

References

[1] Edgar R, Domrachev M, Lash AE. Gene Expression Omnibus: NCBI gene expression and hybridization array data repository Nucleic Acids Res. 2002 Jan 1;30(1):207-10

[2] Pedraza V, Gomez-Capilla JA, Escaramis G, Gomez C et al. Gene expression signatures in breast cancer distinguish phenotype characteristics, histologic subtypes, and tumor invasiveness. Cancer 2010 Jan 15;116(2):486-96.

Index

∗Topic **EntropyExplorer, differential entropy, differential coefficient of variation, differential expression**
EntropyExplorer, 1
∗Topic **breast cancer, gene expression, microarrays**
GEO BreastCancer, 5

BreastCancer *(*GEO BreastCancer*)*, 5

EntropyExplorer, 1

GEO BreastCancer, 5
